# Accuracy of Genomic Prediction for Foliar Terpene Traits in *Eucalyptus polybractea*

**DOI:** 10.1534/g3.118.200443

**Published:** 2018-06-11

**Authors:** David Kainer, Eric A. Stone, Amanda Padovan, William J. Foley, Carsten Külheim

**Affiliations:** * Research School of Biology, The Australian National University, Acton ACT 2601, Australia; † Center for BioEnergy Innovation, Biosciences Division, Oak Ridge National Laboratory, Oak Ridge, TN 37831

**Keywords:** terpenes, essential oil, *Eucalyptus*, genomic prediction, BLUP|GA, shared data resources, GenPred

## Abstract

Unlike agricultural crops, most forest species have not had millennia of improvement through phenotypic selection, but can contribute energy and material resources and possibly help alleviate climate change. Yield gains similar to those achieved in agricultural crops over millennia could be made in forestry species with the use of genomic methods in a much shorter time frame. Here we compare various methods of genomic prediction for eight traits related to foliar terpene yield in *Eucalyptus polybractea*, a tree grown predominantly for the production of Eucalyptus oil. The genomic markers used in this study are derived from shallow whole genome sequencing of a population of 480 trees. We compare the traditional pedigree-based additive best linear unbiased predictors (ABLUP), genomic BLUP (GBLUP), BayesB genomic prediction model, and a form of GBLUP based on weighting markers according to their influence on traits (BLUP|GA). Predictive ability is assessed under varying marker densities of 10,000, 100,000 and 500,000 SNPs. Our results show that BayesB and BLUP|GA perform best across the eight traits. Predictive ability was higher for individual terpene traits, such as foliar α-pinene and 1,8-cineole concentration (0.59 and 0.73, respectively), than aggregate traits such as total foliar oil concentration (0.38). This is likely a function of the trait architecture and markers used. BLUP|GA was the best model for the two biomass related traits, height and 1 year change in height (0.25 and 0.19, respectively). Predictive ability increased with marker density for most traits, but with diminishing returns. The results of this study are a solid foundation for yield improvement of essential oil producing eucalypts. New markets such as biopolymers and terpene-derived biofuels could benefit from rapid yield increases in undomesticated oil-producing species.

Breeding and selection in long-lived tree species face several challenges that reduce gain per unit time. First, it can take years for progeny to reach maturity, which extends the cycle time for selection of mature traits. Second, estimation of breeding values with the traditional best linear unbiased prediction (BLUP) animal model (Henderson 1984) is reliant on pedigree information to describe the genetic covariance between individuals. The pedigrees of relatively undomesticated tree populations are often shallow compared to those available in annual crops and domesticated livestock. Furthermore, in open-pollinated species such as White spruce (*Picea glauca*) (Beaulieu *et al.* 2014) or Loblolly pine (*Pinus taeda*) (Zapata-Valenzuela *et al.* 2013) the relationships between progeny may be incorrectly assigned (Isik 2014), resulting in lower accuracy of estimated breeding value (EBV). Finally, many important quantitative traits are highly polygenic with numerous QTL of mostly small effect, so marker assisted selection (MAS) is not useful because too many loci need to be tracked in the breeding population (Holland 2004; Isik 2014). Genomic selection (GS), however, is an alternative to marker-assisted selection that can address these issues.

In GS the additive genetic effects of genome-wide markers (*e.g.*, SNPs) are jointly estimated so that an individual’s breeding value can be predicted solely from their genotype (Meuwissen *et al.* 2001). Initially a training population has both their phenotype and genotype assessed in order to develop the genomic prediction model. The model is then applied to the genotypes of un-phenotyped individuals in order to estimate their breeding values. Since individuals can be genotyped at a young age, selection on genomically estimated breeding value (GEBV) can be performed before the mature trait is observable, resulting in reduced cycle time and often greater gain (Wong and Bernardo 2008; Grattapaglia and Resende 2011; Kumar *et al.* 2012). GS can also improve the accuracy of estimated breeding values by capturing accurate relationships at genome-wide SNPs, or by directly modeling the genetic architecture of the trait. The errors and assumptions from pedigree-based BLUP are therefore corrected by GS (Munoz *et al.* 2014). Due to its advantages over traditional methods, GS is becoming increasingly prevalent in plant and tree breeding. Some recent examples include White spruce (Beaulieu *et al.* 2014; Ratcliffe *et al.* 2017), *Eucalyptus* (M. D. V. Resende *et al.* 2012; Durán *et al.* 2017; Tan *et al.* 2017), and Loblolly pine (M. F. R. Resende *et al.* 2012; Zapata-Valenzuela *et al.* 2013). For traits such as wood density, height, diameter and growth, GS has consistently matched or exceeded the accuracy and gain from pedigree BLUP. To date, however, no study has investigated the use of GS for improving the yield of tree-sourced essential oils, such as Eucalyptus oil sourced from the leaves of various eucalypt species.

*Eucalyptus polybractea* (blue mallee) is the primary species grown for commercial production of Eucalyptus oil in Australia due to its high foliar oil concentration of up to 13% of dry weight and desirable oil composition (King *et al.* 2006; Kainer *et al.* 2017). The oil is a mixture of mostly monoterpenes, fewer sesquiterpenes and other volatile and non-volatile organic compounds. The monoterpene 1,8-cineole is the primary constituent that gives the oil its desired characteristics. The market for pure Eucalyptus oil is niche with less than 10,000 tons produced annually, but there is increasing interest in larger scale industrial uses for specific terpenes found in the oil, such as for biofuels, polymers and solvents (Mewalal *et al.* 2017). Currently, most *E. polybractea* plantations are established from open-pollinated seed sources that were selected from native stands for their relatively high oil concentration and high proportion of 1,8-cineole. As a consequence, much of the existing commercial stock is undomesticated, resulting in inconsistent yields between years and lines (Goodger and Woodrow 2012). Due to great natural variation there is much scope for improvement in the traits that are important for large scale production of plant-based terpenes: foliar oil concentration, 1,8-cineole proportion, leafy biomass accumulation, growth and survivability (Doran and Matheson 1994; Grant 1997; Doran 2002; Kainer *et al.* 2017). Genomic selection holds the promise of rapid domestication of *E. polybractea* and other oil producing species (Kainer *et al.* 2015), though it is worth evaluating the genomic predictive ability for oil traits relative to traditional methods.

The accuracy of genomic prediction is affected by the genetic architecture of the trait, the choice of model, and the density of markers available to the model. Most oil and growth traits are quantitative and are likely to be highly polygenic, with genetic architectures that may be captured more accurately by some models than others. At the same time the outcrossing undomesticated nature of *E. polybractea* suggests very short-range linkage disequilibrium LD within the population (Thumma *et al.* 2005), requiring high density markers to ensure that all relevant quantitative trait loci (QTL) are tagged by at least one marker. Here we make use of high density SNPs derived from whole genome resequencing of the study population, allowing a comparison of predictive ability when using both high and low SNP densities within multiple model frameworks.

As a base model we employ Genomic BLUP (GBLUP), a simple and robust GS approach that is commonly used in forestry for calculating GEBVs (Zapata-Valenzuela *et al.* 2013). While traditional ABLUP models use the assumed relationship between individuals from a pedigree, GBLUP exchanges the pedigree-based relationship matrix in the ABLUP animal model for a realized genomic relationship matrix (GRM) derived from marker genotypes (VanRaden 2008). GBLUP is computationally efficient with large numbers of SNPs and often provides better accuracy than traditional BLUP (Su *et al.* 2014). However, a shortcoming of GBLUP is that each genotyped SNP is assumed to explain an equally small (and non-zero) proportion of the total genetic variance, which is biologically unrealistic and may reduce GEBV accuracy if the trait’s true genetic architecture departs considerably from that assumption (Daetwyler *et al.* 2010; de Los Campos *et al.* 2013; Bernardo 2014). In reality, when genome-wide SNPs are available, the majority of SNPs probably have negligible effect on the trait, some will have a small effect and a few (if any) will have a larger effect (Meuwissen *et al.* 2001). To address this we also use the BayesB model which assumes that a proportion, π, of the SNPs are in regions with no effect while 1-π SNPs are in LD with causative loci (or are causative themselves). For trait architectures that contain loci of moderate to large effect BayesB often improves accuracy over GBLUP, as demonstrated in a moderately sized population of loblolly pine (Gao *et al.* 2015).

A shortcoming of BayesB is that it requires the user to preselect values for priors like π and the inverse Chi-squared distribution scale and degrees of freedom. While several more complex Bayesian models have been developed to estimate these priors in a locus-specific manner from the data at hand (Gianola 2013), Gao *et al.* (2015) showed that BayesB was still often superior for population sizes below 500. In this study our population size was relatively small, so rather than use the more complex Bayesian models, we elected to use an approach known as BLUP|GA (“*BLUP given Genetic Architecture*”), which allows for the use of locus-specific weightings within the computationally efficient GBLUP framework (Zhang *et al.* 2014; Zhang *et al.* 2015). In previous testing, BLUP|GA outperformed GBLUP and more computationally demanding Bayesian models in almost every scenario, including six traits in Loblolly pine.

This is the first study to assess the accuracy of genomic prediction for eight foliar terpene related traits relative to using traditional pedigree-based prediction in a population of 480 *E. polybractea* derived mostly from natural populations. We aim to provide groundwork for using genomic selection to rapidly domesticate such species to produce higher yields of essential oils or for specific terpenes of interest. The results presented here may lead to greater gains in plant-based terpene production, which will ensure future supply of increasingly important renewable products.

## Materials and Methods

Full details of the field arrangement, tree measurements, oil extraction, GC-MS protocol and phenotypic data analysis can be found in Kainer *et al.* (2017). A short description of the field site and phenotyping is provided below.

### Field site

We selected twelve trees from each of 40 open pollinated half-sibling families (N = 480) in a progeny trial at the property of GR Davis Ltd, West Wyalong, Australia. The mother trees for 37 of the families are located in natural stands of *E. polybractea* in the surrounding West Wyalong region and were selected for high essential oil and 1,8-cineole concentration. The mother trees for the remaining three families are located in a first-generation seed orchard at the property. In the progeny trial, each half-sib family plot is planted as a double row of approximately 600 trees running in the East-West direction, with the family plots parallel to each other separated by 3 m. Within each family plot we sampled the twelve trees from only the Western end to minimize within-family environmental variance.

### Phenotypes

We sampled fresh mature leaf in ethanol containing 0.25 g L^-1^ of n-tetradecane for extraction of essential oil and subsequent GC-MS analysis to determine the concentration of individual terpenes 1,8-cineole (CIN) and α-pinene (APIN), the total concentration of all monoterpenes (MONO), the total concentration of all sesquiterpenes (SESQ), and the total essential oil concentration (OC). SESQ and APIN were square-root and log transformed, respectively. The proportion of total oil that is 1,8-cineole (PCIN) was also calculated as 1,8-cineole is the dominant terpene in *E. polybractea* and PCIN is a key trait for the quality determination of pharmaceutical-grade Eucalyptus oil. Tree height (HT) was measured in the field when the trees were one year post-coppice (March 2013) and two years post-coppice (March 2014). The growth trait (dHT) represents the one year absolute change in height. Due to within-family environmental variation, a postblocking factor was introduced which assigned individuals to one of four blocks in the East-West direction. Phenotypes were adjusted for the fixed effect of postblock.

### Genotypes

We used genotypes obtained from low coverage whole genome re-sequencing (WGS) in a genome-wide association study (GWAS) of the same population (D. Kainer, A. Padovan, W.J. Foley, C. Külheim, unpublished data). Briefly, to obtain genotypes we extracted DNA from frozen leaf collected from all 480 trees using the Qiagen DNeasy Plant kit (Qiagen, Valencia, CA, USA) and then prepared barcoded libraries for Illumina sequencing using a modified version of the protocol by Rohland and Reich (2012). Libraries were sequenced with 125 bp paired-end sequencing on the Illumina HiSeq 2500 platform. After QC and demultiplexing the sequenced reads, 468 out of the original 480 libraries were considered to have adequate sequencing coverage for genomic analyses.

We aligned the reads from the highest depth sample in each family to the *Eucalyptus grandis* reference genome (Myburg *et al.* 2014) using BWA-mem (Li and Durbin 2009) and called variants with Freebayes (Garrison and Marth 2010). We then took high confidence single nucleotide variants that were fixed for an alternate allele in our population relative to the reference and replaced those sites in the reference with the alleles from our population. This produced an *E. polybractea* ‘pseudo-reference’ to which we aligned all 468 samples for final variant calling. Due to the overall low depth of coverage (most samples were between 3-5x depth genome-wide) we used the Thunder variant calling pipeline (Li *et al.* 2011). Thunder is able to make use of the LD inherent to the family structure in the population to improve genotype accuracy at low depth sites. After genotyping, we removed variants with less than 0.95 average genotype confidence, very low (< 800) or very high (> 3200) total depth, or within 5 bp distance to INDELs.

The WGS genotype data contains over 2.3 m SNPs, with the vast majority having low minor allele frequency (MAF). SNPs with very low MAF may introduce errors in genomic prediction since there is an increased chance that they do not segregate in both the training and validation population. Therefore we filtered out SNPs with MAF < 0.05. To evaluate the effect of marker density on predictive ability, we produced three SNP sets with progressively fewer markers. The first set, 500K, contains approximately 502,000 SNPs with MAF > 0.05 after removing SNPs with pairwise LD *R*^2^ > 0.05 within a 2 SNP sliding window using the SNPRelate R package (Zheng *et al.* 2012). The second set, 100K, contains approximately 97,000 SNPs with MAF > 0.05 after removing SNPs from the 500K set with pairwise LD *R*^2^ > 0.05 within an 8 SNP sliding window. The third set, 10K, contains approximately 10,000 SNPs with MAF > 0.05 after removing SNPs from the 100K set with pairwise LD *R*^2^ > 0.05 within a 30 SNP sliding window.

### Prediction and cross-validation

We tested the accuracy of each model by dividing the total population of 468 into training and validation sets using randomized sixfold cross validation (CV). Each fold of cross-validation used 390 individuals as the training set while the remaining 78 had their phenotypes masked for use as the validation set. Accuracy of genomic selection is ideally measured as the correlation between genomic estimated breeding value (GEBV) and true breeding value (TBV) (Garrick *et al.* 2009) in the validation set. Since there is no deep pedigree in *E. polybractea* to provide reliable approximations of TBVs, we instead calculated the predictive ability for each CV iteration as the Pearson’s correlation between the GEBVs and the phenotype adjusted for postblock fixed effects, r(GEBV, y_adj_), of the individuals of the validation set (Daetwyler *et al.* 2013).

To test the effect of SNP density on predictive ability we performed GS using each of the three SNP datasets (10K, 100K, 500K). For every trait and SNP density (8 traits × 3 densities = 24 scenarios), we performed 10 replicates (reps) of sixfold cross-validation per model. Within each replicate, every individual was placed in a validation set exactly once so that every individual had its GEBV estimated once per rep. The predictive ability of a rep is the mean r(GEBV, y_adj_) across the six cross-validations within that rep, and we present the result for each model in each scenario as the mean predictive ability for the 10 reps.

### Pedigree BLUP (ABLUP)

We used the traditional animal model BLUP (Henderson 1984) to establish a benchmark for predictive ability in related individuals which had not been phenotyped. The model is of the form:

y=Xb+Za+e (1)

Where y is a vector of phenotypes, *b* is the vector of postblock fixed effects to be estimated, *a* is a vector of additive genetic random effects to be estimated (*i.e.*, BLUPs of breeding values), *X* is the design matrix relating individuals to fixed effects, *Z* is the design matrix relating individuals to additive genetic effects, and *e* is the vector of individual residual error random effects. The distribution of *a* ∼ N(0, *A*σ_a_) where *A* is the pedigree relationship matrix and σ_a_ is the total additive genetic variance, while *e* ∼ N(0, *I*σ_e_) where *I* is the identity matrix and σ_e_ is the residual error variance. Equation 1 was solved using REML by the *mixed.solve* function in the R package *rrBLUP* v4.4 (Endelman 2011) in order to predict the pedigree-based breeding values for each individual in the validation set.

### Genomic BLUP (GBLUP)

We used GBLUP as the default genomic prediction model. GBLUP uses the same general equation as model (1) but with the pedigree matrix *A* replaced by the realized genomic relationship matrix *G*. The *G* matrix was calculated from genotype data as:

$$\text{G}=\frac{{\text{MIM}}^{T}}{\underset{i}{\sum }2p_{\text{i}}(1-p_{i})}$$ (2)

where *I* is the identity matrix, *M* is a matrix of marker genotypes of dimensions *m* individuals x *n* markers, and *p_i_* is the minor allele frequency of the *i^th^* marker. The genotype of each individual at each marker is represented by {-1,0,1} where 0 is heterozygous, -1 and 1 are the opposing homozygotes. We calculated *G* with the *cpgen v0.2* R package (Heuer 2016). We then predicted GEBVs for the individuals in the validation sets using the *kin.blup* function in the R package *rrBLUP*.

### BayesB

We used the R package VIGoR 1.0 (Onogi and Iwata 2016) to estimate breeding values in cross-validation individuals with a variational Bayesian regression approach. Though BayesB requires a preset of priors, such as π and the parameters for the inverse chi squared distribution from which SNP-effects are drawn, VIGoR provides a mechanism for tuning these hyperparameters and estimating others. For each trait and SNP density a range of hyperparameter priors were permuted (see Table 1) and used to calculate the mean-squared error (MSE) within the CV folds of one rep. The set of hyperparameters resulting in the lowest MSE was then used to estimate breeding values using CV in all 10 reps.

**Table 1:** VIGoR parameters used for to tune BayesB hyperparameters

VIGoR Parameter	explanation	Prior values
**Nu**	Inverse-chi-squared df	4, 8, 12
**Kappa**	Proportion of SNPs with effect > 0 (1-π)	0.05, 0.01, 0.001
**f**	Inbreeding coefficient	0.10
**Mvar**	Proportion of phenotypic variance explained by SNPs	0.50

### BLUP|GA (weighted GBLUP)

Whereas GBLUP disregards trait architecture by treating every marker equally, both BayesB and BLUP|GA attempt to pre-select and up-weight those markers that explain a non-negligible proportion of the trait variance, though BLUP|GA does so within the efficient GBLUP framework.

With BLUP|GA a subset of markers, *M_S_*, is selected from the full genotype matrix *M*, where *M_S_* contains SNPs that are assumed to define the genetic architecture of the trait (*i.e.*, explain much of the trait’s genetic variance). *M_S_* will be weighted so that the genotypes of the selected SNPs influence the calculation of genetic covariance differently to all SNPs in *M*. The selection of SNPs in *M_S_* can be informed by different information sources, such as estimated marker effects from a GWAS, or by a-priori candidate gene and functional biology studies. *M_S_* and *M* are then used to construct two GRMs, *S* and *G* respectively, which are recombined into one trait-specific GRM, known as the *T*-matrix, for use in a standard GBLUP model:

T=ωS+(1−ω)G (3)

where *G* is a standard GRM constructed from all SNPs per equation 2; *S* is a GRM constructed from only the *M_S_* SNPs, per equation 4, with the identity matrix (I) used in equation 2 replaced by *D*, a diagonal matrix, which contains SNP-specific weightings:

$$\text{S}=\frac{{\text{M}}_{\text{S}}{\text{DM}}_{\text{S}}^{\text{'}}}{\underset{i}{\sum }2p_{\text{i}}(1-p_{i})}$$ (4)

BLUP|GA provides two mechanisms for weighting the selected SNPs: i) the genomic architecture weighting, ω, provides an easily adjusted mechanism that allows for coarsely varying the overall importance of *S* during the construction of the *T* matrix. The impact of *S* (and hence the *M_S_* SNPs) can vary from ω = 0 which is the equivalent of standard unweighted GBLUP, to ω = 1 which applies maximum weighting to the *S* matrix and is the equivalent to using only the *M_S_* SNPs in the construction of the GRM. We tested the predictive ability of BLUP|GA for 0.0 < ω < 1.0 in increments of 0.1, noting that ω = 0 is the equivalent of GBLUP; ii) D provides a fine-scaled weighting mechanism for the SNPs within *M_S_* by allowing for varying importance of individual SNPs during the construction of the *S* matrix, and ultimately the *T* matrix. To keep *S* on the same scale as *G*, diag(*D*) is normalized to have a mean of one.

We selected *M_S_* and defined *D* by estimating SNP effects with BayesB using only the genotypes and phenotypes of the individuals from the training set in each cross validation fold. Hyperparameters were chosen to shrink the vast majority of SNP effects toward zero, thus exaggerating the effect of SNPs that are likely to truly be a part of the genetic architecture. We set π = 0.999, implying only 0.1% of SNPs have an effect. SNP effects were drawn from an inverse chi-squared distribution with 4 degrees of freedom and a scale parameter of 0.01, which implies that the vast majority SNP effect sizes are very small with a few being considerably larger. We then selected the SNPs comprising *M_S_* based on whether their squared estimated effect (*µ*^2^) was in the largest 0.1% of effect sizes (Tiezzi and Maltecca 2015). We also included in *M_S_* the SNPs immediately flanking each of the selected SNPs to account for SNPs most likely to be in LD with the selected SNPs, and then generated the weights for diag(*D*) using the squared estimated effects of those selected SNPs.

### Data Availability

The genotype data, phenotype data and R code used in this study are available on FigShare at https://figshare.com/s/be57a3a4d49742dd6fdf. We implemented BLUP|GA in R (R Core Team 2017) and have made the functions available as a package at https://github.com/dkainer/BLUPGA.

## Results

### Pedigree kinship

**Figure 1:**
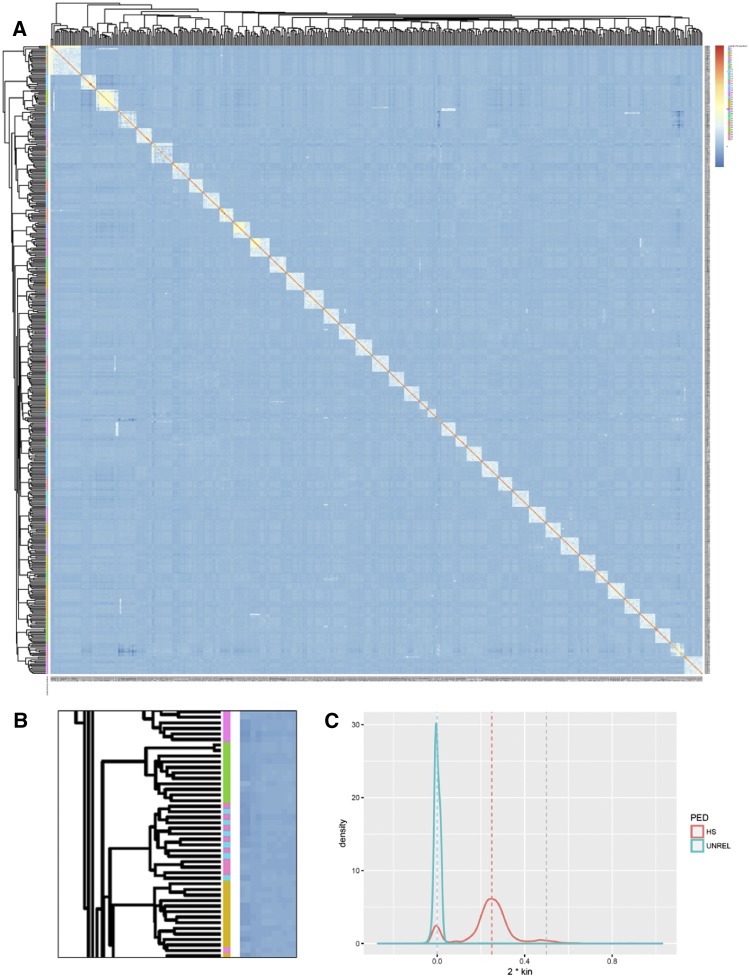
Genomic relatedness between individuals and families - A) heatmap of clustered pairwise kinship. Darker blue indicates zero kinship (*i.e.*, unrelated), red indicates kinship of one (*i.e.*, self or clonal) while intermediate values show varying levels of relatedness. Each individual is annotated on the y-axis with a color according to its pedigree-assigned family. B) Magnification of a section of the y-axis. Contiguous color bars demonstrate genomic relationship clustering in concordance with the pedigree. Striping indicates that the family assignment in the pedigree was mixed for two families. C) Most pedigree-based half-sib (HS) pairs show kinship in a normal distribution around the expected value of 0.25 (orange line main peak), though a number of assumed pedigree half-sibs are actually unrelated or full-sib, as indicated by their distribution around 0 and 0.50 respectively.

A clustered heatmap of the genomic relationship matrix (GRM) produced from 100K SNPs shows that the assumed pedigree of 40 half-sib families was mostly recovered from the genotype data (Figure 1A). That is, hierarchical clustering of pairwise kinship distances recovered 25 out of the 40 half-sib families completely, and another 7 families had all but one of their members clustered together correctly. The heatmap also revealed possible pedigree errors from the field, with some families showing completely mixed genomic membership (Figure 1B). A number of assumed half-sibs were, in fact, unrelated and some assumed half-sibs show full-sib relatedness (Figure 1C).

### Genomic prediction

For each trait we performed 10 reps of sixfold cross-validation (CV) in each of the three SNP density scenarios (10K, 100K, 500K). In each CV we calculated the predictive ability of ABLUP, GBLUP, BayesB and BLUP|GA. The mean predictive ability for a rep is the mean r(GEBV, y_adj_) of the sixfold CV. In Table 2 the mean predictive ability of the 10 reps is presented for each trait, scenario and model.

**Table 2:** Mean predictive ability of each model in each trait for three SNP densities (NSNP).

TRAIT	TYPE	NSNP	ABLUP	GBLUP	BayesB	BLUPGA^#^
**OC**	OIL	10K	0.278	0.333	0.353	0.339
**OC**	OIL	100K	0.278	0.360	0.375	0.362
**OC**	OIL	500K	0.278	0.368	0.380	0.368
**MONO**	OIL	10K	0.276	0.374	0.401	0.390
**MONO**	OIL	100K	0.276	0.398	0.417	0.403
**MONO**	OIL	500K	0.276	0.406	0.422	0.407
**SESQ**	OIL	10K	0.444	0.438	0.459	0.439
**SESQ**	OIL	100K	0.444	0.465	0.475	0.539
**SESQ**	OIL	500K	0.444	0.469	0.473	0.497
**CIN**	OIL	10K	0.335	0.688	0.705	0.703
**CIN**	OIL	100K	0.335	0.701	0.724	0.711
**CIN**	OIL	500K	0.335	0.706	0.727	0.716
**APIN**	OIL	10K	0.302	0.552	0.566	0.553
**APIN**	OIL	100K	0.302	0.563	0.574	0.573
**APIN**	OIL	500K	0.302	0.567	0.576	0.587
**PCIN**	OIL	10K	0.398	0.770	0.764	0.771
**PCIN**	OIL	100K	0.398	0.784	0.787	0.785
**PCIN**	OIL	500K	0.398	0.783	0.787	0.790
**HT**	BIOMASS	10K	0.084	0.197	0.187	0.247
**HT**	BIOMASS	100K	0.084	0.161	0.169	0.196
**HT**	BIOMASS	500K	0.084	0.160	0.163	0.172
**dHT**	BIOMASS	10K	0.108	0.136	0.132	0.192
**dHT**	BIOMASS	100K	0.108	0.134	0.133	0.182
**dHT**	BIOMASS	500K	0.108	0.141	0.140	0.167

# BLUP|GA with weighting of the top 0.1% of SNPs by squared effect size as estimated with BayesB. BLUP|GA includes the case of *ω*=0 in each CV, so the minimum possible outcome for each CV is equal to GBLUP.

The predictive ability of breeding values estimating using the animal model (ABLUP) were poor compared to the predictive ability of GEBVs. For some traits, such as CIN, PCIN and HT, predictive ability was as low as half that of the genomic models. The only trait where ABLUP was competitive was SESQ, though the genomic models were still considerably better when high SNP densities were available to them.

The predictive ability of GEBVs was generally higher for oil traits than for biomass traits, as expected due to the generally higher heritability of oil traits (Table 2). Aggregate oil traits such as MONO (the concentration of all monoterpenes), SESQ (the concentration of all sesquiterpenes) and OC (the overall concentration of all terpenes) were less predictable than major single terpene concentrations CIN (1,8-cineole) and APIN (α-pinene). Notably 1,8-cineole, which is the dominant terpene in *E. polybractea* foliar oil, had both its foliar concentration (CIN) and its proportion of total oil (PCIN) very accurately predicted by all models tested. Mean predictive ability was as high as 0.727 for CIN and 0.787 for PCIN using BayesB.

**Figure 2:**
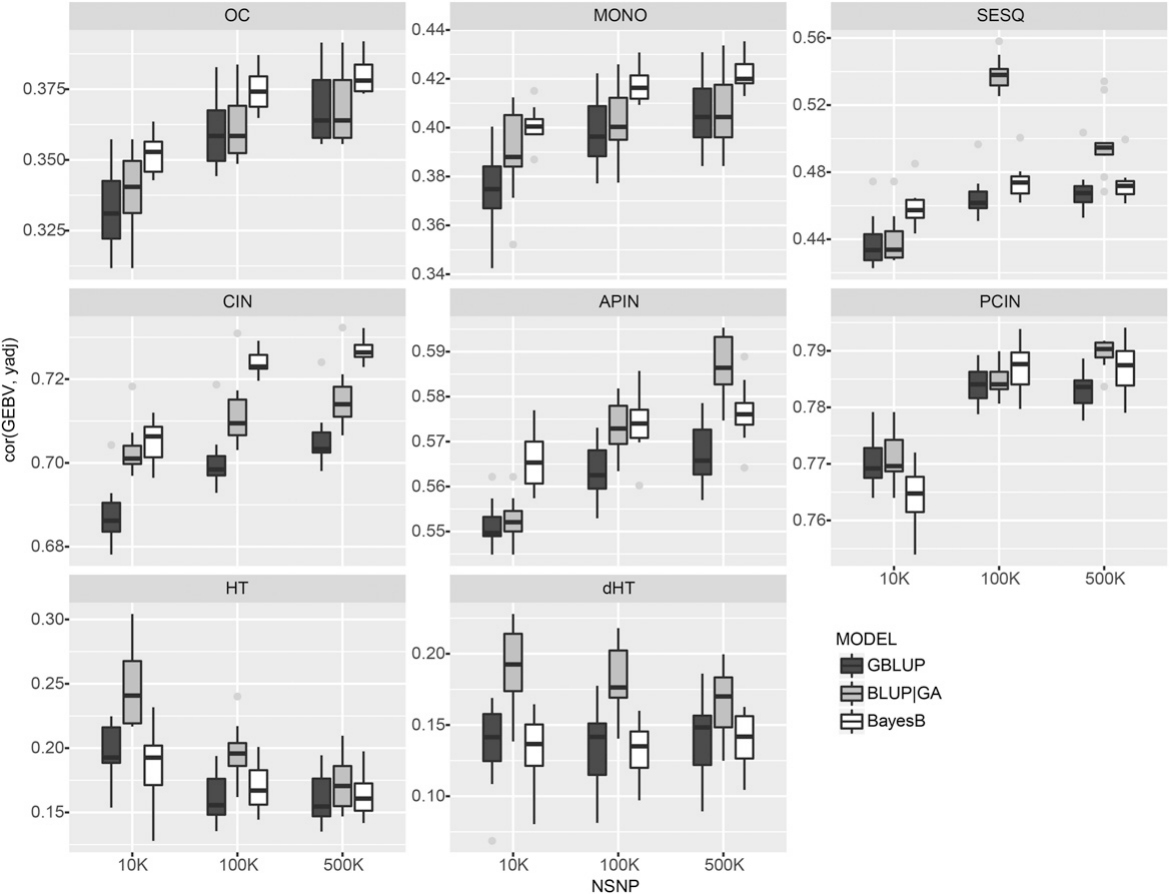
Predictive ability for GBLUP, BayesB and BLUP|GA. Boxplots show the distribution of mean predictive ability from 10 reps of sixfold cross validation. GBLUP, BLUPGA and BayesB results are shown in dark, medium and light gray respectively.

BayesB was superior to GBLUP in 19/24 scenarios tested, and was particularly strong for prediction in oil traits rather than biomass traits (Figure 2). BayesB was the most accurate model in 13/18 scenarios for oil traits while BLUP|GA was most accurate for the remaining 5 scenarios for oil traits. For oil traits, BayesB provided a 2.9% median relative gain in predictive ability over GBLUP. BLUP|GA, on the other hand, was the most accurate model for all scenarios of biomass traits (HT and dHT), and provided a 22.6% median relative gain in predictive ability over GBLUP.

**Figure 3:**
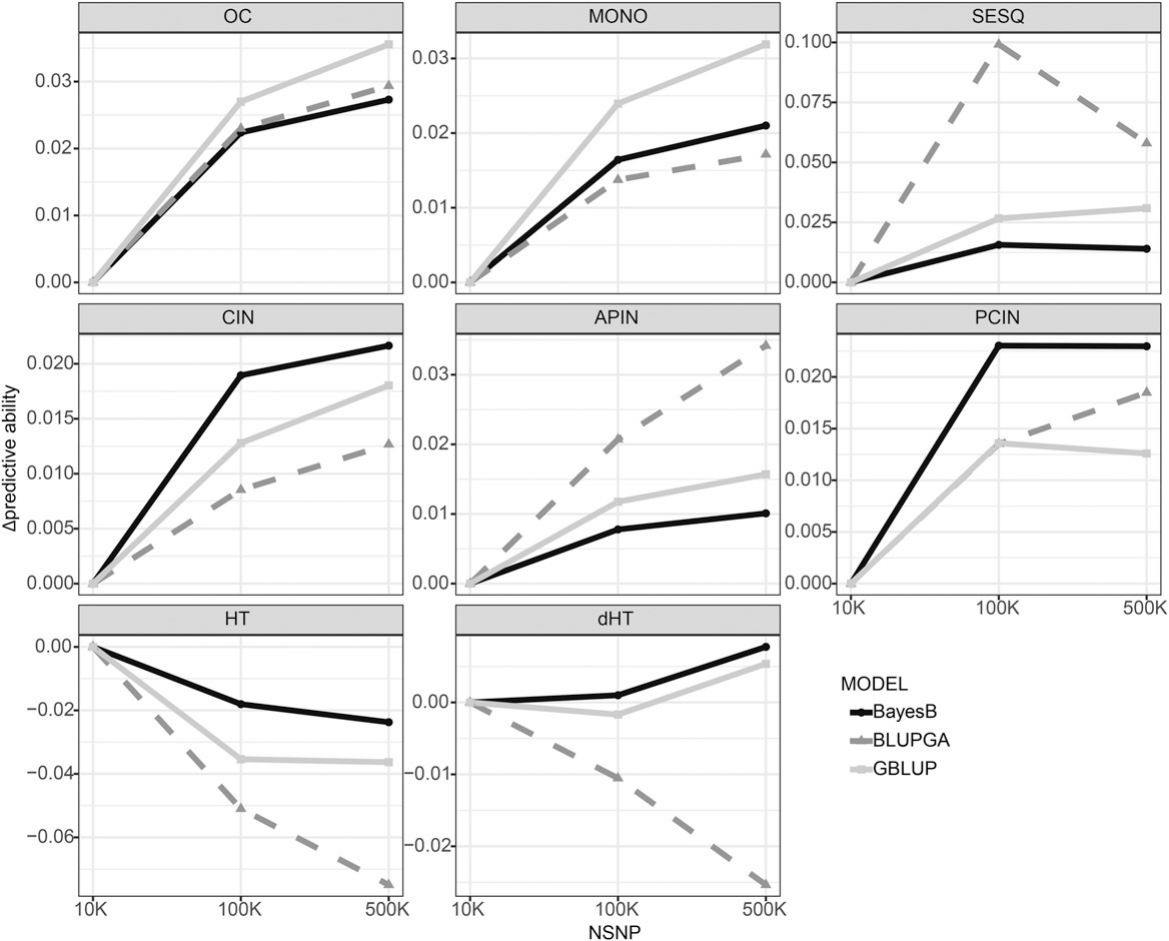
Effect of increasing SNP density on predictive ability for oil and biomass traits. The result obtained with 10K SNPs is used as the baseline. Points at 100K and 500K SNPs represent the absolute difference in predictive ability compared to the predictive ability with 10K SNPs. GBLUP is show in light gray, BLUP|GA in dark gray and BayesB in black.

Increasing the SNP density from 10K to 100K resulted in improved predictive ability for all oil-related traits regardless of the model used (Figure 3). Further increasing SNP density from 100K to 500K resulted in additional gains in predictive ability when using GBLUP or BayesB, however the gains were considerably less than those from 10K to 100K, indicating that increased SNP density provided by whole genome sequencing may provide diminishing returns. For biomass traits, increased SNP density from 10K to 100K surprisingly appeared to have a neutral or negative effect on predictive ability.

Increasing SNP density occasionally had a stronger effect for BLUP|GA than it did on GBLUP or BayesB. In the most drastic instance, BLUP|GA predictive ability for SESQ increased from 0.439 to 0.539 when NSNP increased from 10K to 100K (Figure 3).

**Figure 4:**
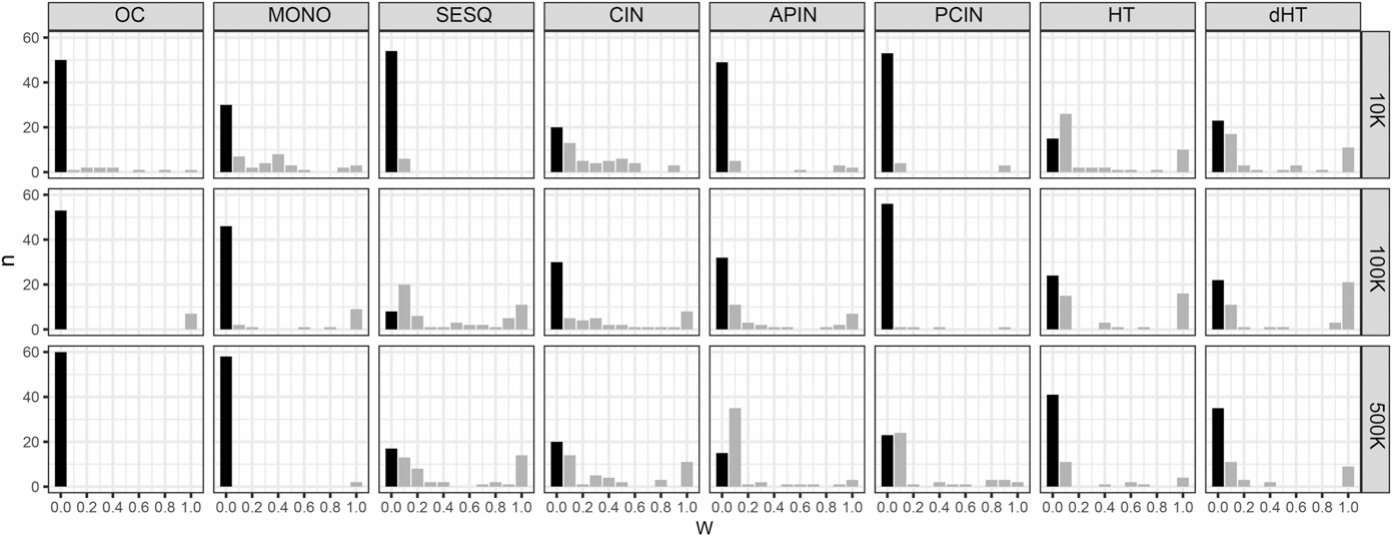
Frequency of the value of ω for which BLUP|GA predictive ability was highest. By increasing ω for 0.0 ≤ ω ≤ 1.0 in increments of 0.1, each CV fold provided a measure of predictive ability when varying importance is placed on the weighted SNPs and the unweighted SNPs (see equation 3). If the maximum predictive ability is achieved at ω=0 this is equivalent to GBLUP, and is shown here by dark bars. When maximum predictive ability is achieved at ω>0 (light bars) the SNP selection and weighting procedure has improved the model over GBLUP for that CV fold. Each row shows the results at different SNP densities (10K, 100K, 500K).

The ability for BLUP|GA to improve predictive ability over GBLUP by weighting selected SNPs appears to be very dependent on the trait and SNP density available. The predictive ability of BLUP|GA for a given trait and SNP density can be summarized based on three possible outcomes from cross-validation: i) maximum predictive ability was achieved at ω = 0. This occurs when the weighting of selected SNPs provides no benefit to the model so the model falls back to standard GBLUP. Black bars in Figure 4 show the frequency of this outcome; ii) maximum predictive ability was achieved at 0.0 < ω < 1.0. This means that the use of weighted SNPs does increase predictive ability over standard GBLUP, depending on how weakly or strongly the weightings are applied in equation 3; iii) maximum predictive ability was achieved at ω = 1. This means that the weighted SNPs provide the greatest improvement in predictive ability over GBLUP when used entirely on their own to construct the GRM without any input from the remaining SNPs in the dataset. Across all traits and SNP densities, maximum predictive ability of BLUP|GA was achieved at ω = 1 in 10.5% of CVs. In 57.9% of CVs the maximum predictive ability of BLUP|GA was no better than that of GBLUP (*i.e.*, at ω = 0).

For OC, BLUP|GA very rarely achieved its maximum predictive ability at ω > 0, which means that the process of weighting the top 0.1% of SNPs according to their estimated effect sizes was usually detrimental to predictive ability compared to standard GBLUP. For CIN and SESQ, on the other hand, BLUP|GA very often achieved maximum predictive ability at ω > 0, indicating that the weighting of SNPs was more useful for these traits if the appropriate SNPs were available. In fact, in 14/60 CV iterations run for SESQ using 500K SNPs, the maximum BLUP|GA predictive ability achieved was at ω = 1 (Figure 4).

## Discussion

Forest products have been utilized by human civilizations for millennia, but unlike many agricultural products such as corn and wheat, tree species have not undergone similar extensive domestication processes. In the 21^st^ century, forestry may hold some solutions to current modern world problems and may mitigate climate change. Trees may provide traditional building products as well as renewable feedstocks to replace proportions of fossil fuel-based feedstocks for new products, such as cellulose-based polymers and advanced biofuels. Many tree species exhibit large phenotypic variation of desirable traits and while this is promising to tree improvement, long maturation and generation times have slowed traditional breeding processes. The use of molecular markers and genomic information has already begun to increase yield gains per unit time in forest species. Here we utilize, for the first time, genomic prediction as a tool for improvement of essential oil yield in the oil producing tree species *Eucalyptus polybractea*.

Traditional breeding in forest species uses known pedigree data to calculate estimated breeding values with the best linear unbiased prediction model (equation 1). However, pedigree information in forestry species is often shallow, incomplete or partially incorrect due to human error or unintended cross-pollination. Here the importance of using genomic data for the estimation of breeding values in forest tree systems is immediately apparent from the kinship analysis which reveals considerable pedigree error within this progeny trial population. It is somewhat difficult to pinpoint whether the pedigree error occurred in the silvicultural operation (*e.g.*, during the initial establishment of family plots) or is partly due to mis-labeling in the sample handling and processing for this study. Nevertheless, the pedigree error is probably the primary cause of the poor performance of traditional ABLUP for the prediction of breeding values seen here. The genomic prediction models, which either correct the pedigree errors using SNP data or estimate GEBVs from marker effects, generally perform much better than ABLUP and, if implemented in a breeding program, would result in far greater gain per cycle for key oil-yield traits such as 1,8-cineole concentration (CIN) and proportion (PCIN).

### Predictive ability for oil and growth traits

For the Eucalyptus oil market, key traits of interest include total oil concentration (OC), proportion of 1,8-cineole (PCIN) and 1,8-cineole concentration (CIN). The ability for coppiced mallees to regrow vigorously is also important. Our results show moderate predictive ability of 0.38 for OC, and very high predictive ability up to 0.73 and 0.79 for CIN and PCIN, respectively. This is highly promising for breeders looking to develop high 1,8-cineole producing lines. Post-coppice height growth (dHT), though less predictable (0.24), may still be a useful trait for multi-trait selection. However, one must be wary of selecting strongly for height since rapid vertical growth can result from biomass accumulation in the stems rather than leaf, which is detrimental to oil yield (Kainer *et al.* 2017). The predictive ability for leaf:stem ratio and total leaf biomass would be worth investigating.

### Factors affecting genomic prediction accuracy

Pérez-Enciso *et al.* (2015) demonstrated through simulation that in a perfect scenario where every causal SNP was known a-priori, a trait with variance controlled by 100 QTNs could be predicted using just those 100 SNPs with an accuracy of 0.95. Using more broad (but accurate) biological knowledge of the trait architecture, such as all SNPs residing in causal genes, still gave a large boost in accuracy over using all available WGS SNPs in the model. Thus, for a given trait, if we can use a model with a set of SNPs that provides the most accurate representation of genetic architecture, then we can progress toward the ideal case of Pérez-Enciso *et al.*, with great benefits for breeders. In reality, for quantitative traits where we do not know which SNPs or genes are causal, prediction accuracy is subject to the density of markers and the ability of the model to successfully isolate relevant SNPs from noisy background. Such is the case in this study, so we assessed predictive ability with three different model approaches and three SNP densities derived from WGS.

Essential oil-yield has a complex genetic trait architecture, which we have thus far only partially unraveled. Previous work led to the assumption that most of this trait is controlled through the biosynthetic pathway that produces terpenes (Külheim *et al.* 2011; Webb *et al.* 2013; Padovan *et al.* 2014; Webb *et al.* 2014). However, eucalypts are among the few taxa which store essential oils in schizogenic leaf cavities (Ishizaki 2015) and work by King and colleagues (2006) found a strong correlation between leaf cavity volume and oil concentration, indicating that genes involved in the leaf ontogeny and cavity formation may play a part in controlling oil yield. Other factors that may contribute to the genetic architecture of oil yield include terpene transport, storage and precursor availability and competition (Vickers *et al.* 2014). Considering all of these factors, there may be a large number of small effect QTL underpinning the variation in terpene concentration and proportion, and even more so for aggregate traits such as total oil concentration (OC).

We found that for most oil traits BayesB outperformed BLUP|GA, which in turn outperformed GBLUP, though most of the time the differences in predictive ability were fairly small (Table 2). BayesB is at its most powerful when there are a few QTL of moderate to large effect in the trait architecture (Daetwyler *et al.* 2010), so its inability here to improve greatly over GBLUP supports the hypothesis that traits like oil concentration and 1,8-cineole concentration are highly polygenic with mostly small-effect QTL. It is likely that BayesB has limited power to isolate the relevant SNPs of small effect size due to the relatively small population in the study.

**Figure 5:**
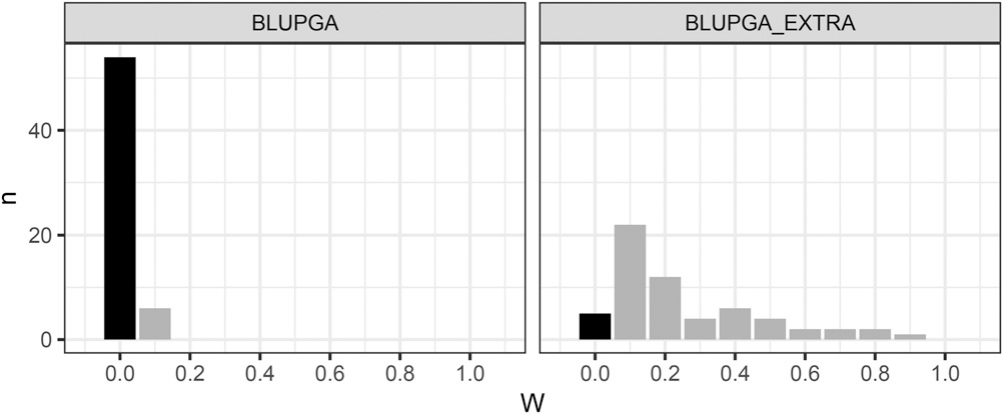
BLUP|GA for SESQ using 10K SNPs with and without a key large effect SNP. BLUP|GA_EXTRA refers to the results of BLUP|GA for the 10K SNP set with one extra SNP of large effect added. This large effect SNP was found in the 100K and 500K SNP sets, but not in the 10K set. Without the extra SNP, maximum BLUP|GA predictive ability occurred at ω = 0 (*i.e.*, GBLUP) in most CV iterations (left, dark bar). The inclusion of the single extra SNP causes the SNP weighting process to have a highly positive effect on predictive ability, with the vast majority of CV iterations having maximum predictive ability at ω > 0 (right, light bars).

BLUP|GA, while more accurate than GBLUP, was less accurate than BayesB for most oil traits. The exception was for total sesquiterpenes (SESQ). At low SNP density (10K), the maximum predictive ability achieved for SESQ by BLUP|GA was 0.439. However, with a 10-fold increase in SNP density BLUP|GA showed greatly increased predictive ability of 0.539. On investigating why increasing the SNP density resulted in such a major improvement of BLUP|GA for SESQ, we noted that a specific SNP of large effect on chromosome 10 was present in the 100K SNP set but was not present in the 10K SNP set. We then explicitly added this SNP to the 10K set, forming the 10K_EXTRA SNP set. Without the extra SNP, BLUP|GA achieved its maximum predictive ability at ω > 0 in only 6/60 cross-validations, indicating almost no positive effect from weighting selected SNPs. However, with the extra SNP, the maximum predictive ability achieved now occurred at ω > 0 in 55/60 cross-validations, showing that weighting this single SNP in the GRM had a major impact (Figure 5). This indicates that much of the benefit of weighting SNPs in the BLUP|GA GRM comes from SNPs that tag loci with a large effect on the trait, which agrees with the observations of Zhang *et al.* (2015). If such SNPs are missing from the genotype data, then the trait architecture is not accurately represented and BLUP|GA may fail to provide any increase in accuracy over GBLUP. Thus it is not the increase in SNP numbers *per se*, but the increase in tagged QTL that produces benefits from higher density genotyping.

Given the presence of a large-effect locus for SESQ on chromosome 10, it is perhaps surprising that BayesB performed only marginally better than GBLUP and considerably worse than BLUP|GA when given the same 100K SNP data. After all, the strength of BayesB is in selecting SNPs with moderate to large effect. Gao *et al.* (2015) showed that the need to manually set data-wide priors such as π, rather than estimate them in a locus-specific manner from the data, is a limitation for BayesB. In this case, the BayesB priors used may be sub-optimal for the architecture of SESQ, resulting in a failure to adequately capture the causal SNPs. BLUP|GA, on the other hand, is able to make use of relatedness information carried by thousands of neutral or near-zero effect SNPs in addition to the genetic architecture information provided by the weighted SNPs of larger effect. This provides two lines of information for predictive purposes. Daetwyler *et al.* (2012) demonstrated that the accuracy of genomic prediction can indeed be decomposed into components due to 1) relatedness between individuals and 2) SNP-QTL LD, and encouraged the use of models that access both branches of information. The authors of that study also noted that relatedness comprised the greater proportion of accuracy. Considering the strong presence of family relatedness in our study it is not surprising that BLUP|GA is generally unable to improve on GBLUP for polygenic traits where individual SNP effects are hard to estimate accurately. In such a scenario the weighting of SNPs is unlikely to be correctly applied, thus producing a weighted GRM which accurately reflects neither true relatedness nor trait architecture. When increasing importance is given to the weighted GRM (*i.e.*, as ω approaches 1.0), the outcome is accuracy below that of GBLUP. Indeed, Pérez-Enciso *et al.* (2015) noted that using partially incorrect biological prior information, such as weighting SNPs from both causal and non-causal genes, has a negative impact on prediction accuracy relative to using all SNPs in an unweighted manner. Therefore, it is worth further investigation of oil-yield traits through GWAS to improve our understanding of their genetic architecture. This will provide a basis for more accurate biological priors for genomic prediction methods of essential oil traits.

### WGS genotyping

The utility of whole genome sequencing (WGS) for genomic prediction has been debated in recent years. The question reflects a balance between the extra cost of sequencing the entire genome of each individual (as opposed to employing GBS or SNP chip) and the potential improvement in accuracy due to higher marker density. While simulations (Meuwissen and Goddard 2010; Druet *et al.* 2014; Pérez-Enciso *et al.* 2015) and empirical studies (Ober *et al.* 2012; Tan *et al.* 2017) have shown that increased marker density leads to higher GEBV accuracy, the accuracy often rapidly reaches a plateau. Others have shown that the WGS markers have no benefit over chip genotypes, or may even make SNP effect estimation more error prone and therefore introduce error into the GS models (Pérez-Enciso *et al.* 2015; Heidaritabar *et al.* 2016). In this study the 10K SNP set represents an approximation of the number and spread of SNPs likely to be obtained through genotyping this population with the *Eucalyptus* EUChip60k SNP chip (Silva-Junior *et al.* 2015). We see that increased SNP density from 10K up to 500K generally results in increased predictive ability for most models and traits tested, but with diminishing returns. The gain in predictive ability from 10K to 500K was moderate for most traits and was in fact negative for height, which would seem to imply that chip-based genotyping may be more cost-effective than WGS. However, the decision to use WGS would still appear justified based on the great improvement in predictive ability of the BLUP|GA model between the 10K SNP set and larger SNP sets for sesquiterpene concentration. Due to the very short LD in this undomesticated, out-crossing population, the 10K SNP density is potentially insufficient to tag important QTL, should they exist. Furthermore, an additional 1–5% in GEBV accuracy from WGS genotyping can have significant economic impact in breeding perennials where selections have long term consequences. When taking into account the ever-decreasing cost of high throughput sequencing, WGS would appear to provide the best chance for maximizing GEBV accuracy across a wide variety of traits in many non-model crops.

### Conclusions

Genomic prediction can be used to accurately guide selection for high essential oil producing individuals, and in particular for those with high concentrations and proportions of major terpenes, such as 1,8-cineole. Compared to traditional BLUP, genomic models provide greater predictive ability due to their ability to avoid the pitfalls of incorrect pedigree specification, which is a problem in undomesticated species such as *E. polybractea*. Genotyping approach and model selection are key factors that can influence the predictive ability, sometimes by a considerable amount. In turn these are subject to the genetic architecture of the traits of interest. Greater understanding of trait architecture through GWAS methods may help to improve predictive ability further, with positive implications for both essential oil and terpene production.

## References

[bib1] Beaulieu J. Doerksen T. K. Clément S. MacKay J. Bousquet J., 2014 Accuracy of genomic selection models in a large population of open-pollinated families in white spruce. Heredity 113: 343–352. 10.1038/hdy.2014.36 24781808 PMC4181072

[bib2] Bernardo R., 2014 Genomewide selection when major genes are known. Crop Sci. 54: 68–75. 10.2135/cropsci2013.05.0315

[bib3] Daetwyler H. D. Calus M. P. L. Pong-Wong R. de Los Campos G. Hickey J. M., 2013 Genomic prediction in animals and plants: Simulation of data, validation, reporting, and benchmarking. Genetics 193: 347–365. 10.1534/genetics.112.147983 23222650 PMC3567728

[bib4] Daetwyler H. D. Kemper K. E. van der Werf J. H. J. Hayes B. J., 2012 Components of the accuracy of genomic prediction in a multi-breed sheep population. J. Anim. Sci. 90: 3375–3384. 10.2527/jas.2011-4557 23038744

[bib5] Daetwyler H. D. Pong-Wong R. Villanueva B. Woolliams J. A., 2010 The impact of genetic architecture on genome-wide evaluation methods. Genetics 185: 1021–1031. 10.1534/genetics.110.116855 20407128 PMC2907189

[bib6] de Los Campos G. Hickey J. M. Pong-Wong R. Daetwyler H. D. Calus M. P. L., 2013 Whole-genome regression and prediction methods applied to plant and animal breeding. Genetics 193: 327–345. 10.1534/genetics.112.143313 22745228 PMC3567727

[bib7] Doran J. C., 2002 Genetic improvement of eucalypts: With special reference to oil-bearing species in Eucalyptus: The genus Eucalyptus, Taylor & Francis, London.

[bib8] Doran J. C. Matheson A. C., 1994 Genetic parameters and expected gains from selection for monoterpene yields in Petford *Eucalyptus camaldulensis*. New For. 8: 155–167. 10.1007/BF00028191

[bib9] Druet T. Macleod I. M. Hayes B. J., 2014 Toward genomic prediction from whole-genome sequence data: impact of sequencing design on genotype imputation and accuracy of predictions. Heredity 112: 39–47. 10.1038/hdy.2013.13 23549338 PMC3860159

[bib10] Durán R. Isik F. Zapata-Valenzuela J. Balocchi C. Valenzuela S., 2017 Genomic predictions of breeding values in a cloned *Eucalyptus globulus* population in Chile. Tree Genet. Genomes 13: 74 10.1007/s11295-017-1158-4

[bib11] Endelman J. B., 2011 Ridge Regression and other kernels for genomic selection with R Package rrBLUP. Plant Genome 4: 250–255. 10.3835/plantgenome2011.08.0024

[bib12] Gao N. Li J. He J. Xiao G. Luo Y., 2015 Improving accuracy of genomic prediction by genetic architecture based priors in a Bayesian model. BMC Genet. 16: 120 10.1186/s12863-015-0278-9 26466667 PMC4606514

[bib13] Garrick D. J. Taylor J. F. Fernando R. L., 2009 Deregressing estimated breeding values and weighting information for genomic regression analyses. Genet. Sel. Evol. 41: 55 10.1186/1297-9686-41-55 20043827 PMC2817680

[bib14] Garrison, E., and Marth, G., 2010 Haplotype-based variant detection from short-read sequencing. *arXiv* 1207.3907. https://arxiv.org/abs/1207.3907v2

[bib15] Gianola D., 2013 Priors in whole-genome regression: the Bayesian alphabet returns. Genetics 194: 573–596. 10.1534/genetics.113.151753 23636739 PMC3697965

[bib16] Goodger J. Q. D. Woodrow I. E., 2012 Genetic determinants of oil yield in *Eucalyptus polybractea* R.T. Baker. Trees (Berl.) 26: 1951–1956. 10.1007/s00468-012-0744-1

[bib17] Grant, G. D., 1997 *Genetic variation in Eucalyptus polybractea and the potential for improving leaf oil production*. Thesis. Canberra, ACT: Australian National University, Australian National University.

[bib18] Grattapaglia D. Resende M. D. V., 2011 Genomic selection in forest tree breeding. Tree Genet. Genomes 7: 241–255. 10.1007/s11295-010-0328-4

[bib19] Heidaritabar M. Calus M. P. L. Megens H.-J. Vereijken A. Groenen M. A. M. Bastiaansen J. W. M., 2016 Accuracy of genomic prediction using imputed whole-genome sequence data in white layers. J Anim Breed Genet. 3: 167–179. 10.1111/jbg.12199 26776363

[bib20] Henderson C. R., 1984 Applications of linear models in animal breeding, University of Guelph, Guelph, ON.

[bib21] Heuer, C., 2016 cpgen: Parallel Genomic Evaluations. R package https://github.com/cheuerde/cpgen.

[bib22] Holland J. B., 2004 Implementation of molecular markers for quantitative traits in breeding programs—challenges and opportunities 1–13 in *New directions for a diverse planet**:* *Proceedings of the 4th International Crop Science Congress* Regional Institute, Gosford, Australia, www.cropscience.org.au/icsc2004.

[bib23] Ishizaki K., 2015 Development of schizogenous intercellular spaces in plants. Front Plant Sci 6: 497 10.3389/fpls.2015.00497 26191071 PMC4488600

[bib24] Isik F., 2014 Genomic selection in forest tree breeding: the concept and an outlook to the future. New For. 45: 379–401. 10.1007/s11056-014-9422-z

[bib25] Kainer D. Bush D. Foley W. J. Külheim C., 2017 Assessment of a non-destructive method to predict oil yield in *Eucalyptus polybractea* (blue mallee). Ind. Crops Prod. 102: 32–44. 10.1016/j.indcrop.2017.03.008

[bib26] Kainer D. Lanfear R. Foley W. J. Külheim C., 2015 Genomic approaches to selection in outcrossing perennials: focus on essential oil crops. Theor. Appl. Genet. 128: 2351–2365. 10.1007/s00122-015-2591-0 26239409

[bib27] King D. J. Gleadow R. M. Woodrow I. E., 2006 Regulation of oil accumulation in single glands of *Eucalyptus polybractea*. New Phytol. 172: 440–451. 10.1111/j.1469-8137.2006.01842.x 17083675

[bib28] Külheim C. Yeoh S. H. Wallis I. R. Laffan S. Moran G. F., 2011 The molecular basis of quantitative variation in foliar secondary metabolites in *Eucalyptus globulus*. New Phytol. 191: 1041–1053. 10.1111/j.1469-8137.2011.03769.x 21609332

[bib29] Kumar S. Bink M. C. A. M. Volz R. K. Bus V. G. M. Chagné D., 2012 Towards genomic selection in apple (*Malus × domestica* Borkh.) breeding programmes: Prospects, challenges and strategies. Tree Genet. Genomes 8: 1–14. 10.1007/s11295-011-0425-z

[bib30] Li H. Durbin R., 2009 Fast and accurate short read alignment with Burrows-Wheeler transform. Bioinformatics 25: 1754–1760. 10.1093/bioinformatics/btp324 19451168 PMC2705234

[bib31] Li Y. Sidore C. Kang H. M. Boehnke M. Abecasis G. R., 2011 Low-coverage sequencing: implications for design of complex trait association studies. Genome Res. 21: 940–951. 10.1101/gr.117259.110 21460063 PMC3106327

[bib32] Meuwissen T. Goddard M. E., 2010 Accurate prediction of genetic values for complex traits by whole-genome resequencing. Genetics 185: 623–631. 10.1534/genetics.110.116590 20308278 PMC2881142

[bib33] Meuwissen T. Hayes B. J. Goddard M. E., 2001 Prediction of total genetic value using genome-wide dense marker maps. Genetics 157: 1819–1829. 11290733 10.1093/genetics/157.4.1819 PMC1461589

[bib34] Mewalal R. Rai D. K. Kainer D. Chen F. Külheim C., 2017 Plant-derived terpenes: A feedstock for specialty biofuels. Trends Biotechnol. 35: 227–240. 10.1016/j.tibtech.2016.08.003 27622303

[bib35] Munoz P. R. Resende M. F. R. Huber D. A. Quesada T. Resende M. D. V., 2014 Genomic relationship matrix for correcting pedigree errors in breeding populations: Impact on genetic parameters and genomic selection accuracy. Crop Sci. 54: 1115 10.2135/cropsci2012.12.0673

[bib36] Myburg A. A. Grattapaglia D. Tuskan G. A. Hellsten U. Hayes R. D., 2014 The genome of *Eucalyptus grandis*. Nature 510: 356–362. 10.1038/nature13308 24919147

[bib37] Ober U. Ayroles J. F. Stone E. A. Richards S. Zhu D., 2012 Using whole-genome sequence data to predict quantitative trait phenotypes in *Drosophila melanogaster*. PLoS Genet. 8: e1002685 10.1371/journal.pgen.1002685 22570636 PMC3342952

[bib38] Onogi A. Iwata H., 2016 VIGoR: Variational Bayesian Inference for Genome-Wide Regression. J. Open Res. Softw. 4 10.5334/jors.80

[bib39] Padovan A. Keszei A. Külheim C. Foley W. J., 2014 The evolution of foliar terpene diversity in Myrtaceae. Phytochem. Rev. 13: 695–716. 10.1007/s11101-013-9331-3

[bib40] Pérez-Enciso M. Rincón J. C. Legarra A., 2015 Sequence- *vs.* chip-assisted genomic selection: accurate biological information is advised. Genet. Sel. Evol. 47: 43 10.1186/s12711-015-0117-5 25956961 PMC4424891

[bib41] R Core Team, 2017 *R**: A Language and Environment for Statistical Computing*. R Foundation for Statistical Computing, Vienna, Austria. http://www.R-project.org/

[bib42] Ratcliffe B. El-Dien O. G. Cappa E. P. Porth I. Klápště J., 2017 Single-Step BLUP with varying genotyping effort in open-pollinated *Picea glauca*. G3 (Bethesda) 7: 935–942. 10.1534/g3.116.037895 28122953 PMC5345723

[bib43] Resende M. D. V. Resende M. F. R. Sansaloni C. P. Petroli C. D. Missiaggia A. A., 2012 Genomic selection for growth and wood quality in *Eucalyptus*: capturing the missing heritability and accelerating breeding for complex traits in forest trees. New Phytol. 194: 116–128. 10.1111/j.1469-8137.2011.04038.x 22309312

[bib44] Resende M. F. R. Munoz P. R. Resende M. D. V. Garrick D. J. Fernando R. L., 2012 Accuracy of genomic selection methods in a standard data set of Loblolly pine (*Pinus taeda* L.). Genetics 190: 1503–1510. 10.1534/genetics.111.137026 22271763 PMC3316659

[bib45] Rohland N. Reich D., 2012 Cost-effective, high-throughput DNA sequencing libraries for multiplexed target capture. Genome Res. 22: 939–946. 10.1101/gr.128124.111 22267522 PMC3337438

[bib46] Silva-Junior O. B. Faria D. A. Grattapaglia D., 2015 A flexible multi-species genome-wide 60K SNP chip developed from pooled resequencing of 240 *Eucalyptus* tree genomes across 12 species. New Phytol. 206: 1527–1540. 10.1111/nph.13322 25684350

[bib47] Su G. Christensen O. F. Janss L. Lund M. S., 2014 Comparison of genomic predictions using genomic relationship matrices built with different weighting factors to account for locus-specific variances. J. Dairy Sci. 97: 6547–6559. 10.3168/jds.2014-8210 25129495

[bib48] Tan B. Grattapaglia D. Martins G. S. Ferreira K. Z. Sundberg B., 2017 Evaluating the accuracy of genomic prediction of growth and wood traits in two *Eucalyptus* species and their F 1 hybrids. BMC Plant Biol. 17: 110 10.1186/s12870-017-1059-6 28662679 PMC5492818

[bib49] Thumma B. R. Nolan M. F. Evans R. Moran G. F., 2005 Polymorphisms in *Cinnamoyl CoA Reductase* (CCR) are associated with variation in microfibril angle in *Eucalyptus* spp. Genetics 171: 1257–1265. 10.1534/genetics.105.042028 16085705 PMC1456829

[bib50] Tiezzi F. Maltecca C., 2015 Accounting for trait architecture in genomic predictions of US Holstein cattle using a weighted realized relationship matrix. Genet. Sel. Evol. 47: 24 10.1186/s12711-015-0100-1 25886167 PMC4381547

[bib51] VanRaden P. M., 2008 Efficient methods to compute genomic predictions. J. Dairy Sci. 91: 4414–4423. 10.3168/jds.2007-0980 18946147

[bib52] Vickers C. E. Bongers M. Liu Q. Delatte T. Bouwmeester H., 2014 Metabolic engineering of volatile isoprenoids in plants and microbes. Plant Cell Environ. 37: 1753–1775. 10.1111/pce.12316 24588680

[bib53] Webb H. Foley W. J. Külheim C., 2014 The genetic basis of foliar terpene yield: Implications for breeding and profitability of Australian essential oil crops. Plant Biotechnol. 31: 363–376. 10.5511/plantbiotechnology.14.1009a

[bib54] Webb H. Lanfear R. Hamill J. Foley W. J. Külheim C., 2013 The yield of essential oils in *Melaleuca alternifolia* (Myrtaceae) is regulated through transcript abundance of genes in the MEP pathway. PLoS One 8: e60631 10.1371/journal.pone.0060631 23544156 PMC3609730

[bib55] Wong C. K. Bernardo R., 2008 Genomewide selection in oil palm: increasing selection gain per unit time and cost with small populations. Theor. Appl. Genet. 116: 815–824. 10.1007/s00122-008-0715-5 18219476

[bib56] Zapata-Valenzuela J. Whetten R. W. Neale D. B. McKeand S. Isik F., 2013 Genomic estimated breeding values using genomic relationship matrices in a cloned population of loblolly pine. G3 (Bethesda) 3: 909–916. 10.1534/g3.113.005975 23585458 PMC3656736

[bib57] Zhang Z. Erbe M. He J. Ober U. Gao N., 2015 Accuracy of whole-genome prediction using a genetic architecture-enhanced variance-covariance matrix. G3 (Bethesda) 5: 615–627. 10.1534/g3.114.016261 25670771 PMC4390577

[bib58] Zhang Z. Ober U. Erbe M. Zhang H. Gao N., 2014 Improving the accuracy of whole genome prediction for complex traits using the results of genome wide association studies. PLoS One 9: e93017 10.1371/journal.pone.0093017 24663104 PMC3963961

[bib59] Zheng X. Levine D. Shen J. Gogarten S. M. Laurie C. C., 2012 A high-performance computing toolset for relatedness and principal component analysis of SNP data. Bioinformatics 28: 3326–3328. 10.1093/bioinformatics/bts606 23060615 PMC3519454

